# Chronic Eosinophilic Leukemia Presenting as Treatment-Refractory Nodular Scleritis: A Paraneoplastic Autoimmune Syndrome With Multisystem Inflammatory Manifestations

**DOI:** 10.7759/cureus.105414

**Published:** 2026-03-17

**Authors:** Byung C Ji, Thanda Aung, Janelle Castellino, Kanwarpal S Kahlon, Edmund Tsui, Ben J Glasgow

**Affiliations:** 1 Rheumatology, University of California Los Angeles, David Geffen School of Medicine, Los Angeles, USA; 2 Hematology and Oncology, University of California Los Angeles, Los Angeles, USA; 3 Ophthalmology, University of California Los Angeles, Los Angeles, USA; 4 Eye Pathology, Stein Eye Institute Stein Eye Institute, Los Angeles, USA

**Keywords:** chronic eosinophilic leukemia, etv6-syk fusion, fostamatinib, paraneoplastic syndrome, precision medicine, scleritis

## Abstract

We report the first documented case of chronic eosinophilic leukemia (CEL) with ETV6-SYK gene rearrangement manifesting as refractory bilateral nodular scleritis within a broader paraneoplastic autoimmune syndrome. A 25-year-old woman with pre-existing Hashimoto's thyroiditis developed progressive bilateral nodular scleritis with anterior uveitis that proved completely refractory to high-dose corticosteroids, methotrexate, and conventional disease-modifying antirheumatic drugs, along with papilledema and granulomatous rosacea, suggesting systemic inflammation. Comprehensive evaluation revealed persistent marked eosinophilia (peak 15,000/μL), prompting hematological investigation, including a bone marrow biopsy and cytogenetic analysis that confirmed CEL harboring the ETV6-SYK fusion oncogene. Recognition of this molecular target enabled precision therapy with fostamatinib, a spleen tyrosine kinase (Syk) inhibitor, combined with adalimumab, a tumor necrosis factor-alpha (TNF-α) inhibitor, resulting in complete and sustained remission of all inflammatory manifestations over 18 months of follow-up. This case underscores the critical importance of maintaining high clinical suspicion for paraneoplastic autoimmune syndromes in patients with treatment-refractory inflammatory conditions, particularly when accompanied by atypical systemic or unexplained laboratory findings, and demonstrates that molecularly targeted precision medicine approaches can transform treatment outcomes in complex paraneoplastic rheumatic disorders.

## Introduction

Scleritis is a sight-threatening inflammatory condition affecting the sclera, most often associated with systemic conditions, particularly autoimmune conditions, occurring in the context of an immune-mediated systemic inflammatory condition in up to half of affected individuals, such as rheumatoid arthritis or antineutrophil cytoplasmic antibody (ANCA)-associated vasculitis [1]. The autoimmune nature of scleritis is supported by its frequent association with systemic inflammatory diseases and favorable response to immunosuppressive therapy [2].

Paraneoplastic rheumatic disorders represent a rare but clinically significant manifestation of occult malignancy, with inflammatory myopathies, seronegative (rheumatoid factor negative and anti-cyclic citrullinated peptide (CCP) antibody negative) rheumatoid arthritis, and some atypical vasculitides being the most frequently reported paraneoplastic conditions [3]. These manifestations may precede, occur simultaneously with, or follow the diagnosis of malignancy and are a clinical manifestation of occult cancer not directly related to the tumor or metastasis [4].

The recognition of paraneoplastic inflammatory syndromes is crucial for rheumatologists, as these conditions may precede cancer diagnosis by months to years and often present with atypical features that are refractory to conventional immunosuppressive therapy [5]. Treatment often needs to target the underlying etiology rather than the inflammatory manifestation. Over half of the cases of paraneoplastic vasculitis are secondary to hematologic malignancies, including leukemia, lymphoma, and myelodysplastic syndromes [6]. Abnormal hematologic findings such as leukocytosis, abnormal differential counts, and cytopenias warrant a broader differential that includes hematologic malignancies.

We present an exceptional case of chronic eosinophilic leukemia with ETV6-SYK rearrangement manifesting as treatment-refractory bilateral nodular scleritis with multisystem inflammatory involvement, highlighting the diagnostic and therapeutic challenges and the application of molecularly targeted precision medicine to rheumatological practice.

## Case presentation

**Figure 1:**
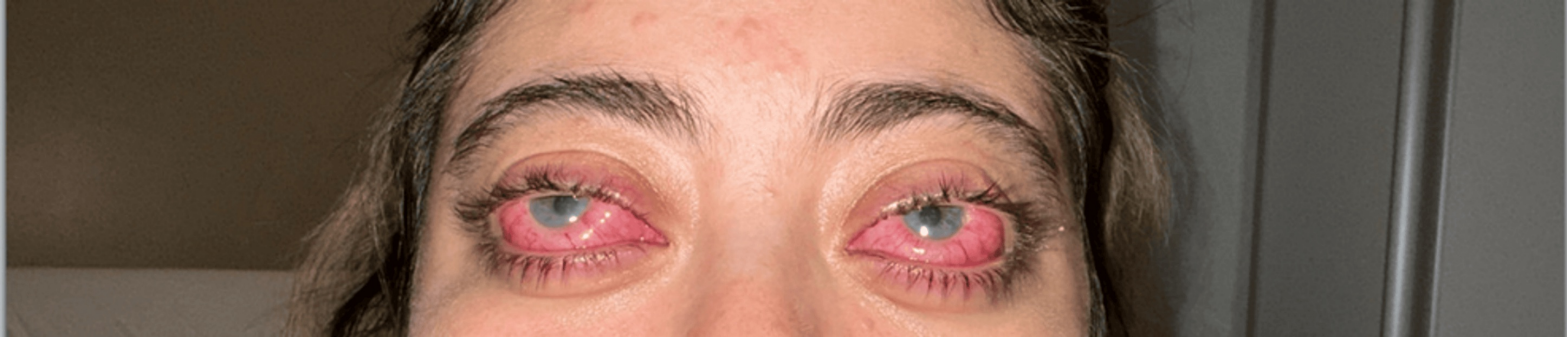
Clinical photographs showing bilateral nodular scleritis with scleral injection, nodular thickening, and conjunctival erythema at presentation.

A 25-year-old woman presented with a several-month history of severe bilateral eye pain, redness, and proptosis as shown in Figure 1. Her medical history was significant for Hashimoto's thyroiditis, suggesting a predisposition to autoimmune disease. Additionally, the bilateral nature and severity of the ocular involvement initially raised suspicion for a systemic inflammatory condition. 

An extensive serologic workup was completed, as detailed in Table 1. Initial rheumatological evaluation revealed a positive antinuclear antibody (ANA) at 1:160 titer with a speckled pattern and positive anti-thyroid peroxidase (TPO) antibodies consistent with her known thyroiditis. Comprehensive autoimmune serological testing, including rheumatoid factor, anti-CCP antibodies, ANCA, anti-double-stranded DNA (dsDNA), complement levels, and extractable nuclear antigens, was negative. However, laboratory studies revealed marked leukocytosis with eosinophilia (absolute eosinophil count 7.34 x 10^3^/uL), which was initially attributed to her history of childhood asthma. Subsequent serologic workup for infectious etiologies, including parasitic causes of eosinophilia, was negative. 

**Table 1:** Laboratory values on presentation

Pertinent lab data	Patient’s lab values	Reference range and units
White blood cell count	20.74	4.16-9.95 x 10^3^/uL
Hemoglobin	13.3	11.6-15.2 g/dL
Hematocrit	39.6	34.9-45.2%
Red blood cell count	4.55	3.96-5.09 x 10^6^/uL
Mean corpuscular volume	87.0	79.3-98.6 fL
Mean corpuscular hemoglobin	29.2	26.4-33.4 pg
MCH concentration	33.6	31.5-35.5 g/dL
Red cell distribution width-SD	45.3	36.9-48.3 fL
Red cell distribution width-CV	14.3	11.1-15.5%
Platelet count, auto	467	143-398 x 10^3^/uL
Mean platelet volume	9.5	9.3-13.0 fL
Absolute neutrophil count	8.90	1.80-6.90 x 10^3^/uL
Absolute monocyte count	0.83	0.20-0.80 x 10^3^/uL
Absolute lymphocyte count	2.65	1.30-3.40 x 10^3^/uL
Absolute basophil count	0.16	0.00-0.10 x 10^3^/uL
Absolute eosinophil count	7.34	0.00-0.50 x 10^3^/uL
Absolute immature granulocyte count	0.86	0.00-0.04 x 10^3^/uL
Sedimentation rate, erythrocyte	45	<=25 mm/hr
Sodium	139	135-146 mmol/L
Potassium	4.5	3.6-5.3 mmol/L
Chloride	106	96-106 mmol/L
Total CO2	22	20-30 mmol/L
Anion Gap	11	8-19 mmol/L
Creatinine	0.66	0.60-1.30 mg/dL
Glucose	93	65-99 mg/dL
TSH	3.6	0.3-4.7 mcIU/mL
Thyroid peroxidase antibody	386	<=20 IU/mL
Thyroid-stimulating immunoglobulin	<0.10	<=0.54 IU/L
C-reactive protein	1.1	<0.8 mg/dL
Antinuclear antibody titer	1:160	<1:40 titer
dsDNA antibody EIA	<=200	<=200 IU/mL
SM antibody	<20	<20 U
RNP antibody	<20	<20 U
SSA antibody	<20	<20 U
SSB antibody	<20	<20 U
Rheumatoid factor	<10	<14 IU/mL
Cyclic citrulline antibody IgG	2	0- 19 units
Beta-2-glycoprotein IgA	<=20	<=20 SAU
Beta-2-glycoprotein IgG	<=20	<=20 SGU
Beta-2-glycoprotein IgM	<=20	<=20 SMU
Cardiolipin IgA	<=20	<=20.0 CU
Cardiolipin IgG	<=20	<=20.0 CU
Cardiolipin IgM	<=20	<=20.0 CU
C-ANCA	<1:20	<1:20 titer
Myeloperoxidase antibody	<20.0	<20.0 CU
P-ANCA	<1:20	<1:20 titer
Proteinase-3 antibody	<20.0	<20.0 CU

**Figure 2:**
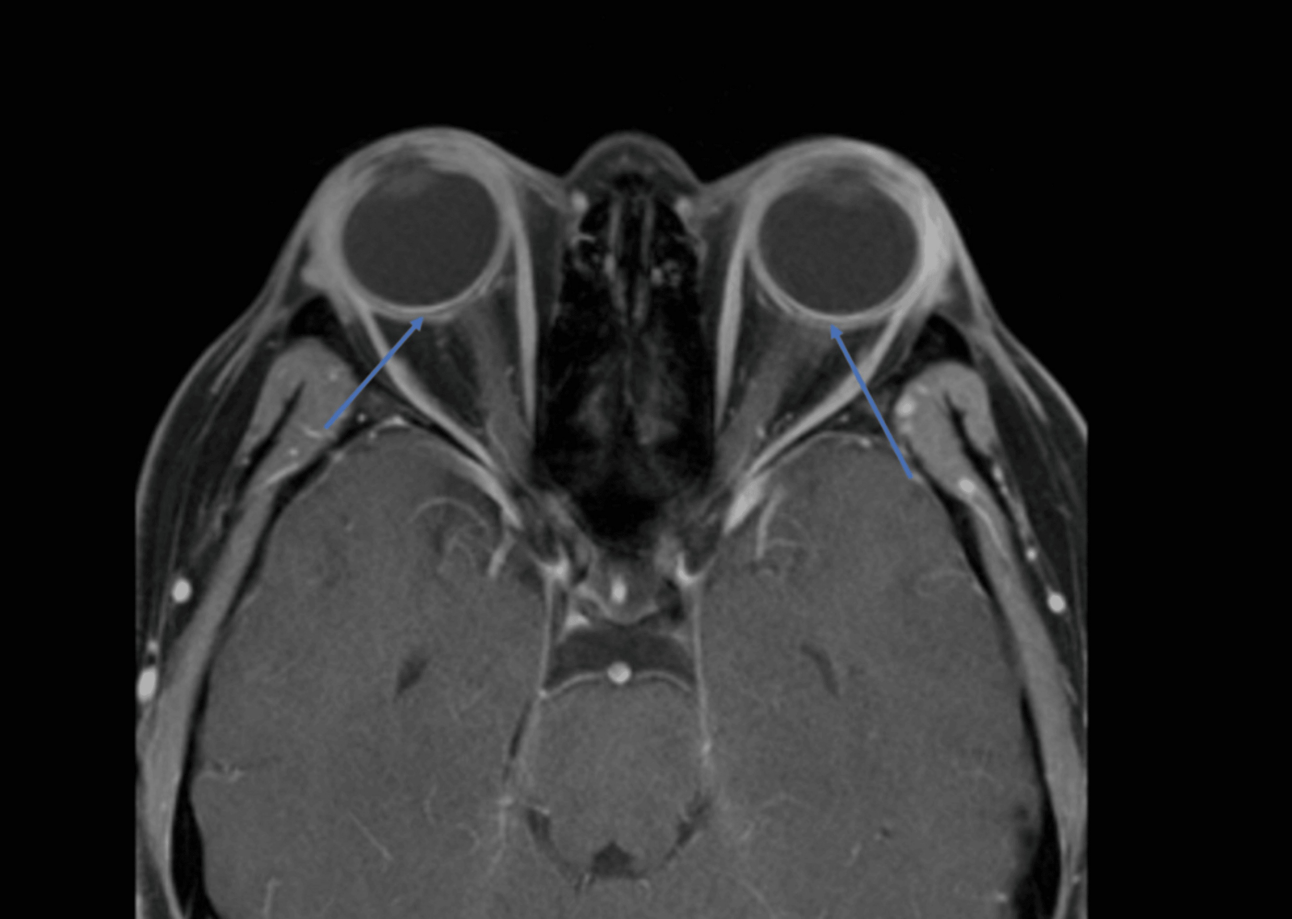
Orbital MRI demonstrating bilateral scleral thickening and enhancement without discrete mass

**Figure 3:**
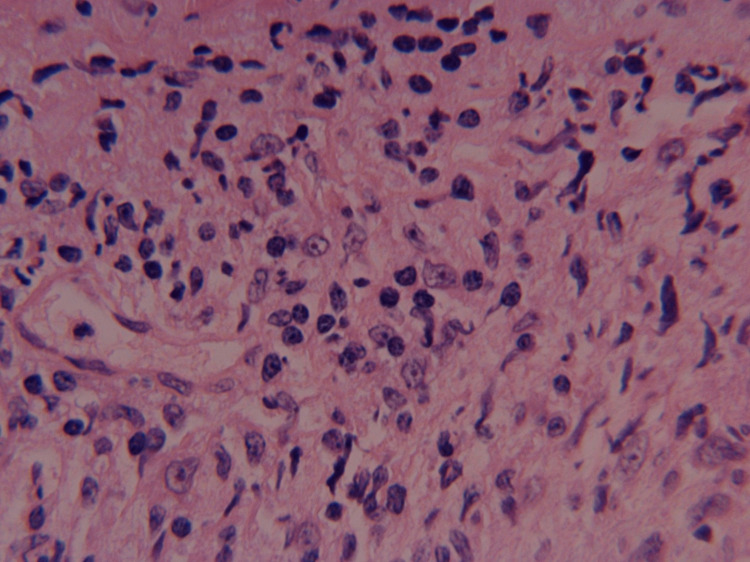
Scleral biopsy histopathology (H&E stain, 40×) demonstrating chronic lymphohistiocytic inflammation with granulomatous features at the scleral border. Note the absence of eosinophilic infiltration despite marked peripheral eosinophilia.

Contrast-enhanced T2-weighted orbital magnetic resonance imaging (MRI) demonstrated bilateral scleral thickening and enhancement, as shown in Figure 2. Scleral biopsy revealed severe chronic lymphohistiocytic inflammation with granulomatous features, absence of eosinophilic infiltration, and negative microbial stains, including periodic acid-Schiff (PAS), Grocott Methenamine Silver (GMS), and acid-fast bacillus (AFB) stains. Immunoglobulin G4 (IgG4) staining was also negative. Corresponding histopathology is shown in Figure 3. The inflammatory pattern was consistent with autoimmune scleritis but lacked the typical eosinophilic infiltrate expected in hypereosinophilic syndromes. 

The patient's condition demonstrated remarkable resistance to conventional anti-inflammatory and immunosuppressive therapies typically effective in autoimmune scleritis. High-dose corticosteroids (prednisone 60 mg daily) tapered over three months resulted in normalization of eosinophil count; however, they provided minimal clinical improvement. The patient was also treated with methotrexate, a cornerstone therapy for many rheumatological conditions and inflammatory eye disease. The patient was treated with 20mg weekly for four months, but was complicated by hepatotoxicity requiring discontinuation. 

During the treatment course, the patient developed additional inflammatory manifestations suggesting systemic involvement. This included papilledema with elevated intracranial pressure (34 mmHg on lumbar puncture), facial erythematous rash subsequently diagnosed as granulomatous rosacea, and a transient Bell's palsy. Neuroimaging was negative for mass effect, venous and sinus thrombosis, and other causes of papilledema. These multisystem inflammatory manifestations, combined with the refractory nature of the disease and persistent eosinophilia, prompted consideration of an underlying systemic process beyond conventional autoimmune disease. 

Bone marrow examination revealed hypercellular marrow with eosinophilia without excess blasts or fibrosis. Fluorescence in situ hybridization (FISH) revealed a loss of one copy of the 3'ETV6-specific signal in 85% (170/200) of nuclei examined, which suggested a partial deletion or unbalanced rearrangement of the ETV6 gene. Next-generation sequencing (NGS) assay detected ETV6-SYK fusion with variant allele frequency (VAF) estimated to be between 40-50%, establishing the diagnosis of CEL with clonal myeloproliferation characterized by ETV6-SYK rearrangement. This molecular finding provided crucial insight into the underlying pathogenesis of the patient's inflammatory syndrome and guided targeted therapeutic intervention. 

Recognition of the ETV6-SYK rearrangement as the driving molecular abnormality led to the simultaneous initiation of fostamatinib, a Syk inhibitor, at 100mg twice daily with adalimumab, a TNF-α antagonist with established efficacy in refractory inflammatory conditions, at 40mg every 14 days. This targeted approach addressed both the primary molecular driver of the malignancy and the downstream inflammatory cascade.

**Figure 4:**
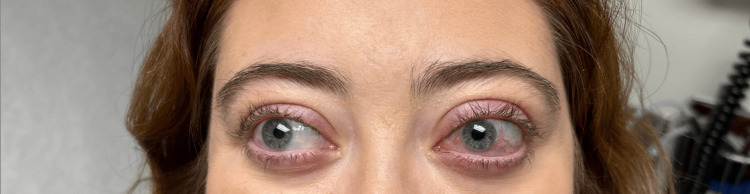
Clinical photograph of improved ocular inflammatory findings after therapy.

The response to targeted therapy was dramatic and sustained. Within one month of initiating combination therapy, the patient experienced significant improvement in scleritis, resolution of papilledema, and clearance of the facial rash as demonstrated in Figure 4. Bone marrow biopsy was repeated after four months of combination therapy. It demonstrated mildly hypocellular marrow with mild eosinophilia. FISH revealed loss of one copy of the 3'ETV6-specific signal in 9% (18/200) of nuclei examined. Fostamatinib was increased to 150mg twice daily, which stabilized her persistent mild eosinophilia. The patient tolerated this combination therapy without adverse effects. By six months, complete remission of all inflammatory manifestations was achieved, with the patient able to discontinue all systemic corticosteroids and return to normal activities. 

## Discussion

This case of refractory scleritis with eosinophilia exemplifies the complex interface between malignancy and autoimmunity that is increasingly recognized in rheumatological practice. Paraneoplastic rheumatic syndromes can precede cancer diagnosis and often present with features that mimic primary autoimmune diseases, making early recognition challenging [3,4].

The patient's presentation with treatment-refractory scleritis accompanied by systemic inflammatory features represents a classic example of paraneoplastic autoimmune syndrome. Several clinical features suggested this diagnosis: the unusual severity and bilateral nature of the scleritis, resistance to conventional immunosuppressive therapy, presence of eosinophilia, and development of multisystem inflammatory manifestations.

While scleritis is classically associated with rheumatoid arthritis and ANCA-associated vasculitis in rheumatological practice, this case highlights the importance of considering broader differential diagnoses. The absence of typical autoimmune serological markers, combined with the refractory nature of the inflammation and presence of eosinophilia, should prompt investigation for underlying hematologic malignancies.

The pathophysiology of scleritis in this context likely involves immune dysregulation involving the SYK signaling and TNF-α pathways secondary to the underlying malignancy rather than direct tissue infiltration. The absence of eosinophils in the scleral biopsy despite marked peripheral eosinophilia supports this hypothesis and suggests that the inflammatory process is mediated by aberrant immune signaling rather than direct cellular infiltration.

The treatment of this patient with targeted therapy and the consequent rapid and sustained remission represents a paradigm shift from traditional immunosuppression to precision medicine approaches in rheumatology. The identification of ETV6-SYK rearrangement provided a rational target for therapy with fostamatinib, while the addition of adalimumab addressed the downstream TNF-α-mediated inflammatory cascade.

This approach contrasts with the conventional "treat-to-suppress" strategy typically employed in autoimmune scleritis and highlights the potential for molecular profiling to guide therapeutic decisions in complex inflammatory conditions. The rapid and sustained response to targeted therapy validates this approach and suggests that understanding underlying molecular mechanisms can lead to more effective treatments with potentially fewer side effects than broad immunosuppression.

Several clinical features in this case should alert rheumatologists to the possibility of paraneoplastic syndrome. These include failure to respond to adequate doses of corticosteroids and conventional disease-modifying antirheumatic drugs (treatment failure), bilateral severe scleritis in a young patient without classic autoimmune serological findings (atypical presentation), persistent eosinophilia not explained by allergic or parasitic causes (laboratory abnormalities), development of multisystem inflammatory manifestations (systemic features), and concurrent presentation of multiple inflammatory conditions (temporal relationships).

This case underscores the importance of multidisciplinary collaboration in managing complex inflammatory conditions. The successful diagnosis and treatment required close coordination between rheumatology, ophthalmology, hematology, and dermatology services. Early recognition of the unusual features and appropriate referral for hematological evaluation were crucial for establishing the correct diagnosis and implementing effective therapy. Ongoing collaboration in monitoring both hematologic and inflammatory responses reinforces the importance of integrated care.

## Conclusions

This case illustrates a unique presentation of CEL manifesting as a paraneoplastic autoimmune syndrome with predominant rheumatological features. It underscores several critical clinical principles for rheumatological practice: first, the necessity of maintaining a high index of suspicion for paraneoplastic syndromes in patients with atypical or treatment-refractory inflammatory conditions, especially those involving lab abnormalities such as eosinophilia. Furthermore, clinicians should broaden their differential diagnosis for inflammatory eye diseases like scleritis beyond traditional autoimmune associations. Ultimately, this case highlights a precision medicine approach-where identified molecular targets can allow for more effective intervention than conventional immunosuppression-and advocates for multidisciplinary collaboration when systemic manifestations span multiple organ systems.

This case contributes valuable insights to the limited literature on paraneoplastic rheumatic syndromes and demonstrates the potential for precision medicine approaches in managing these challenging conditions. Future research should focus on identifying biomarkers that can help distinguish paraneoplastic from primary autoimmune syndromes and developing targeted therapeutic strategies for these rare but clinically significant conditions.
